# Establishment and Verification of Sex- and Age-Specific Serum Electrolyte Reference Intervals in Healthy Han Children in Changchun, Northeastern China

**DOI:** 10.1155/2019/8282910

**Published:** 2019-11-26

**Authors:** Qi Zhou, Xin Li, Yanan Jia, Wenjia Guo, Baojie Guan, Jiancheng Xu

**Affiliations:** ^1^Department of Pediatrics, First Hospital of Jilin University, Changchun 130021, China; ^2^Department of Laboratory Medicine, First Hospital of Shanxi Medical University, Taiyuan 030001, China; ^3^Department of Laboratory Medicine, First Hospital of Jilin University, Changchun 130021, China; ^4^Department of Laboratory Medicine, The General Hospital of FAW, Changchun 130021, China

## Abstract

For lack of feasible interval values from population differences and potential analytical discrepancies, it is essential to ascertain potassium (K), sodium (Na), chlorine (Cl), calcium (Ca), and phosphorus (P) ions reference intervals within Chinese children to fill the gap. Healthy children (*n* = 1391, 2–<15 years old) were recruited from communities and schools to establish sex- and age-specific serum electrolyte reference intervals of Han children in Changchun, China. Levels of serum K, Na, Cl, Ca, and P were measured using a Hitachi 7600-210 automatic biochemical analyzer. Reference intervals were established according to Clinical and Laboratory Standards Institute EP28-A3c guidelines. Data from five representative hospitals located across Changchun were used to verify pediatric serum electrolyte reference intervals. Values were different from adult reference intervals in China. There were sex-specific differences in Na, Cl, Ca, and P reference intervals in 13-<14 children. Serum Na, Cl, and Ca reference intervals showed stable trends within early age groups but fluctuated in teens. Each serum electrolyte had ≤3 age-specific reference intervals. Five laboratories suggested reference intervals were applicable across Changchun.

## 1. Introduction

Clinical interpretation of pediatric medical assessment depends on reliable reference intervals largely which are recognized as “decision supporting tools.” Reference interval is defined as the range between two threshold values, the 2.5 and 97.5 percentiles of results' distribution, within which 95% of observations from healthy individuals fall [1, 2]. Although concept of reference intervals and their application seems quite straightforward and simple, the process of establishing accurate and reliable pediatric reference intervals is complex indeed. Challenges are often caused by scarcity of samples from healthy population, ethical considerations, and discrepancies such as sex- and age-specific differences due to physical size, organ maturity, immune response, and metabolism [1].

Reference intervals in China are from various sources such as industry documents, books, manuscripts, and sometimes manuals [3, 4] currently. Therefore, most values come from decade-old studies among Western populations. Due to ethnic differences, potential discrepancies in analytical procedures, living habit, environment, etc., reference intervals vary among diagnostic laboratories domestically and aboard. With awareness of this discrepancy rising, pediatric serum electrolytes reference intervals are studied in many countries. NORIP (the Nordic Reference Interval Project 2000) [5] has published their results of a similar study; Ridefelt et al. [6] acquired age- and sex-specific serum electrolyte reference intervals for Caucasian population using Abbott Architect platform and suggested that the NORIP reference interval for calcium was too low. Marwaha et al. [7] calculated percentiles of ionized calcium, calcium, and phosphate in detail among healthy 6–17 years old Asian-Indian children and stated they all have shown inverse relationship with age. Furthermore, these data vary greatly even in China. For example, compared with data provided by textbooks (3.5–5.5 mmol/l for K, 130–150 mmol/l for Na, and 94–110 mmol/l for Cl), intervals for adults were 3.6–5.2, 136–146, and 99–110 mmol/l, respectively, according to the Standard of Ministry of Health of the People's Republic of China (2015) [8]; Li et al. [9] established pediatric K reference interval as 3.05–4.46 mmol/L in Huizhou, southern part of China. For now, there is no standard of pediatric serum electrolytes published domestically. Thus, it is essential to ascertain suitable reference intervals within Chinese population.

The Ministry of Health of the People's Republic of China published common biochemical analyte reference intervals of Chinese adults in 2012 [8, 10]. Studies have proved that it was not appropriate to apply adult reference intervals in pediatric population. For instance, serum electrolytes and liver function tests of Han Chinese healthy adult population were found to be different from those of children's [4, 11]. One study found the reference intervals of *N*-terminal probrain natriuretic peptide (NT-proBNP) in the neonatal period (0 to <1 month) and adolescence (13–18 years) were quite different (250.0 to 3987.0  pg/ml vs 20.0 to 145.0  pg/ml) [12]. Age-related differences in reference intervals of children were statistically significant for Ca, Fe, Cu, Mg, and Zn [13]. These research studies demonstrate that inadequate pediatric reference intervals that fail to account for differences between age groups or sex groups lead to misdiagnosis and misclassification of disease. Therefore, it is necessary to establish pediatric reference intervals.

The objective of this study was to establish pediatric serum K, Na, Cl, Ca, and P reference intervals for healthy children in the Han population in Changchun, China. A normal electrolyte balance in the body fluids is necessary for osmoregulation and to maintain nerve and muscle function [14]. Important positively charged ions include K, Na, and Ca, while Cl is a negatively charged ion in serum.

**Figure 1:**
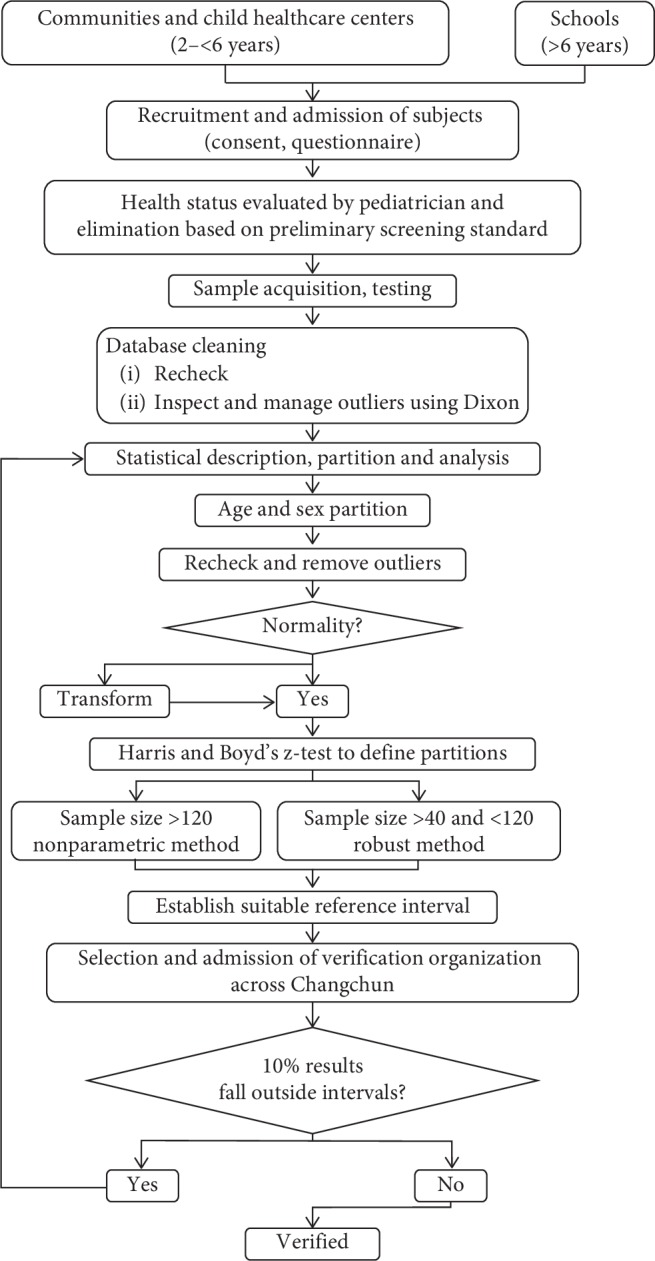
Protocol for establishment and verification of children reference intervals.

We recruited a large number of participants from the Changchun area and followed the procedure for establishing and verifying reference intervals recommended by the Clinical and Laboratory Standards Institute (CLSI) document EP28-A3c [15]. The methods and protocols for this study may be utilized by other regions or countries seeking to establish their own pediatric serum electrolyte reference values (Figure 1).

## 2. Materials and Methods

According to EP28-A3c, the best method to establish a reference interval is to collect samples from a sufficient number of qualified, reference individuals to yield a minimum of 120 samples for analysis for each partition (e.g., sex and age range). However, in the case of difficult-to-obtain subclass reference values for certain populations, such as newborn, pediatric, and geriatric patients, it may be difficult to obtain appropriate age-related reference subjects in sufficient numbers. This study proceeded for 2 years continuously for collecting as many samples as possible for a robust database. Healthy children aged 2 to<15 years were eligible to participate in this study. Children aged 2 to <6 years were enrolled from communities and child healthcare centers in Changchun. Children aged 6 to <15 years were enrolled in primary and middle schools. Finally, a total of 1391 healthy individuals (646 females and 745 males; male: female ratio, 1 : 1.15) were included in this study.

Eligible children or their parents completed a questionnaire that included items pertaining to medical conditions, prescribed and over-the-counter medication, the presence of fever, allergy, or eczema, and general questions about subjective health. Children were excluded from the study if they had (1) a recent history of acute infection, metabolic disease, any systemic disease, use of prescribed medications (within 2 weeks), or surgery (within 6 months) or (2) the following clinical laboratory criteria: positive for HBsAg, HCV, or HIV antibody; creatinine > 120 *μ*mol/L; creatine kinase > 500 U/L; uric acid > 475 *μ*mol/L; glucose > 7.0 mmol/L; or C reactive protein >12.0 mg/L.

This study was approved by the institutional ethics committee of the First Hospital of Jilin University. Written informed consent was provided by all study participants or their parents, and parental permission was obtained prior to collecting serum samples.

Pediatric nurses acquired venous blood from study participants in the community and from child healthcare centers (aged 2–<6 years). Laboratory technicians from the Department of Laboratory Medicine at the First Hospital of Jilin University acquired venous blood from study participants in primary and middle schools (aged 6–<15 years).

During the three days prior to blood collection, all study participants maintained their normal diet and exercise level. Each study participant fasted overnight (>8 hours) before blood was collected in the morning. Samples were collected in a clot-activator tube containing gel (Vacutainer®SST; BD), left at room temperature for 30 min to clot, and centrifuged for 10 mins at 3,000 rpm. Samples that were visually hematolytic, lipemic, or icteric were excluded from the analyses. Samples were received and tested within 2 hours [8, 10].

Serum K, Na, Cl, Ca, and P levels were measured for each study participant. All analyses were performed with a Hitachi 7600-210 automatic biochemical analyzer using the ion-selective electrode method (Hitachi High-Technologies, Tokyo, Japan), according to the manufacturer's instructions and utilizing reagents, calibrators (ISE Standard Low, High and ISE Compensator), and quality control (QC) products (Liquid Assay Multiqual Controls Level 1, Level 2, Level 3; Bio-Rad Laboratories, Inc) provided by the manufacturer. Essential parameters were recorded thoroughly, as lots of reagents and calibrators were replaced every 6 months; verification was performed every time according to CLSI EP26-A guidelines [16]; bias of accuracy (%) and precision were documented according to CLSI EP15-A3 guidelines [17]; carryover rate was tested according to CLSI EP10-A3 guidelines [18]; analytical measurement range was obtained according to CLSI EP17-A2 guidelines [19], clinical reportable range was determined according to CLSI EP 6 guidelines [20].

Samples were analyzed in the Department of Laboratory Medicine at the First Hospital of Jilin University, which was accredited in 2012 according to ISO 15189:2012 Medical Laboratories-Particular Requirements for Quality and Competence by the China National Accreditation Service for Conformity Assessment (CNAS). All clinicians, technicians, and nurses participating in the study had been appropriately trained. The Hitachi 7600-210 automatic analyzer underwent regular maintenance, function checks, calibration, and QC according to the manufacturer's instructions. The analytical performance of the assays, defined as precision, accuracy, analytical measurement range, clinical reportable range, and carryover rate, was carefully monitored [1]. Analytical parameters are presented in Table 1.

The new reference intervals that were established using a Hitachi 7600-210 automatic analyzer in the study participants were verified with different analyzers and in subpopulations living across Changchun. According to EP28-A3c [15], the acceptability of the transfer may be assessed by examining a smaller number of reference individuals (*n* = 20) from the receiving laboratory's own subject population and comparing these reference values to the larger, more adequate original study. Due to the influence of sex and age, the pediatric electrolyte reference interval usually could be divided into several partitioning. For every partitioning, at least 20 healthy individuals covered by age and sex are needed for verification. Verification analyses were conducted according to our previous study in five hospitals located in different areas in Changchun, including Lab 1 (the First Hospital of Jilin University), Lab 2 (the Eastern Division, First Hospital of Jilin University), Lab 3 (the Second Hospital of Jilin University), Lab 4 (the Fourth Hospital of Jilin University), and Lab 5 (the Pediatric Hospital of Changchun) [1]. These laboratories were accredited by the National External Quality Assessment of China. Each laboratory tested samples from the study participants using their hospital's routine instruments, reagents, methods, and controls. The automatic analyzers in Lab 1, Lab 2, Lab 3, Lab 4, and Lab 5 are Hitachi 7600-210 automatic analyzer, Hitachi 7180 automatic analyzer, Hitachi 7180 automatic analyzer, Hitachi 7600 automatic analyzer, and Hitachi 7180 automatic analyzer, respectively. Serum electrolytes from healthy individuals recruited from each of the five hospitals were compared with the reference intervals established in the Department of Laboratory Medicine at the First Hospital of Jilin University. If ≤10% of each subpopulation's results were outside the limits, the established reference intervals were considered applicable [1].

Data analysis was performed as reported in our previous study and in accordance with CLSI EP28-A3c [15] guidelines using EXCEL (Microsoft) and SPSS 21.0 (IBM) [15]. Participants were stratified by sex and classified into 1-year age groups due to uncertainty of logical grouping criterion. For each group, the Dixon test was used to identify outliers, which were removed. Distribution and scatter plots were visually inspected to determine sex and age partitions. One sample Kolmogorov–Smirnov test was used to decide whether a random sample follows a Gaussian distribution. Differences were tested using Harris and Boyd's *z*-test after achieving normality [15], and statistical difference was recognized as *p* < 0.05, which is currently recommended by the CLSI. This test proposes that two groups should be combined to one unless *Z* exceeds *Z*^*∗*^, or the larger standard deviation exceeds the smaller by 1.5 times, regardless of the *Z* values. Lower and upper reference limits are defined as values at the 2.5th and 97.5th percentiles. If one has 120 observations, reference intervals are established by the simple nonparametric approach. For smaller sample sizes, reference intervals are made by the robust approach. Ninety percent confidence intervals were computed for the upper and lower limits of each reference interval [1].

## 3. Results

### 3.1. Characteristics of the Study Participants

A total of 1391 healthy individuals (646 females and 745 males; male: female ratio, 1 : 1.15) were included in this study. Serum K, Na, Cl, Ca, and P levels were measured for each study participant.  Table 2 summarizes the 2.5th and 97.5th percentiles for serum electrolytes stratified by sex and age.

**Figure 2:**
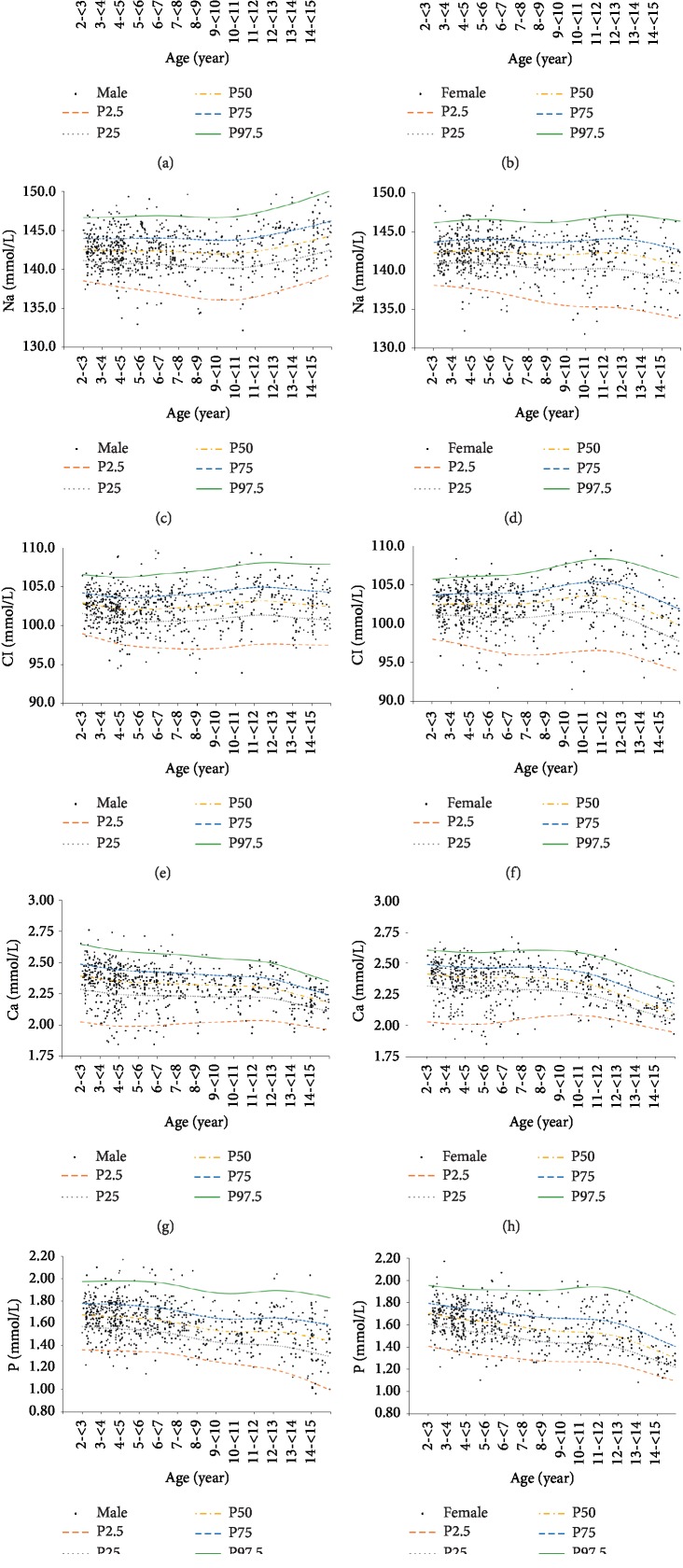
Trends in serum K (a, b), Na (c, d), Cl (e, f), Ca (g, h), and P (i, j) levels in healthy males (a, c, e, g, i) and females (b, d, f, h, j) with age (*n* = 1391). Individual data are presented as dots. P stands for percentile. P2.5 presents as 2.5th value of the group; P25 presents as 25th value of the group; P50 presents as 50th value of the group; P75 presents as 75th value of the group; P97.5 presents as 97.5th value of the group.

Table 3 summarizes sex-specific serum K, Na, Cl, Ca, and P reference intervals in the study participants. There were no significant differences in sex-specific serum K reference intervals in study participants aged 2–<15 years. No significant difference was found between the sexes, with the exception of children aged 13‐14, where serum Na, Cl, Ca, and P reference intervals were higher in males than females (Table 3 and Figure 2).

Study participants were divided into 12 groups by age in one-year gap for 2 to <13 years. There were statistically significant age-specific variations in serum K, Na, Cl, Ca, and P reference intervals. All serum electrolytes required a minimum of 3 age-specific reference intervals. Among these electrolytes, serum Na, Cl, and Ca reference intervals showed a stable trend within the early age groups (Na: 2–<9 y; Ca: 2–<13 y; Cl: 2–11 y) but began to fluctuate in later age groups (Figure 2), whereas serum K and P reference intervals demonstrated complex trends, changing over time. Serum K reference intervals were highest in children aged 2–<4 years and 12–<15 years. Serum P reference intervals were highest in children aged 2 years and lowest in children aged 14 years in both male and female subjects.

### 3.2. Reference Interval Verification

The reference intervals established in our study population were verified in subpopulations recruited in five representative hospitals located throughout Changchun (Table 4). Measurement of serum electrolytes in the subpopulations at the five hospitals revealed all the reference intervals were valid, as no more than 2 of 20 reference values in each subpopulation were outside the reported limits.

## 4. Discussion

Clinical laboratories are responsible for providing appropriate reference intervals of local population. However, there is a distinct lack of pediatric reference intervals in China currently. This study established serum electrolyte reference intervals stratified by sex and arbitrarily classified into groups by age. Actual data were from the results of 1391 Han healthy children aged between 2 and 14 years. Healthy subjects were recruited from community, healthcare centers, primary schools, and middle schools in Changchun.

Our study revealed there were no sex-specific differences among 2 to <15 years in serum K level and 2–<13 years in serum Na, Cl, Ca, and P levels; however, serum Na, Cl, Ca, and P reference intervals were higher in males than females aged 13–<15 years. These may not be consistent with those of others' researches: there were no sex-specific differences in serum K and Na reference intervals among 5–19-year-old children in Denmark [5]; Swedish's [6] data revealed that hardly any sex-specific differences of serum K, Na, Cl, and Ca existed, while difference existed for sure for serum P; CALIPER (Canadian Laboratory Initiative in Pediatric Reference Intervals) reported no sex-specific differences in serum Ca reference intervals among Canadians with difference in serum P level as well [21]. However, it may come from ethnic reasons that Indian boys showed higher serum Ca and P concentrations than counterparts and peaked at ages 6 and 7, respectively [7]. As the second abundant mineral, P functioned as a component in bones and genetic materials, contributing to bipolarity of lipid membranes as well as circulating lipoproteins [22]. A complicated system involving the bones, kidneys, and intestine works together to maintain serum P levels [23]. When it comes to significant changes during puberty, its sex-specific differences might be explained by the sex-dependent alternation in hormonal modulators, including estrogen [24], growth hormone [25], and serum FGF-23 [26]. From physical prospective, estrogen influences serum P with the function of diminishing bone turnover and specifically bone resorption, as claimed by the NHANES (National Health and Nutrition Examination Survey) [24]. Growth hormone (GH) also impacts P through bone remodeling, renal handling, and klotho turnover [25], and its difference between sexes may be the key of P's discrepancy. Fibroblast growth factor 23 (FGF-23), a critical regulator of P homeostasis, was higher in females [26]. Besides, another reason that serum Na, Cl, Ca, and P reference intervals were higher in males from 2 to <13 years could be girls owned a more earlier pubertal development start point.

Researchers revealed that there were significant age-specific differences in serum K, Na, Cl, Ca, and P reference intervals. We proposed all the serum electrolytes required a minimum of 3 age-specific reference intervals. Though trends of K, Na, Cl, and Ca in the present study are not exactly the same as those in Australia [27] and Germany [28], they also need to be divided into several age-related partitions. Similarly, serum P reference intervals in children in India [7], Denmark [5], Canada [29], and Sweden [6] decreased with age. We speculate that this relates to absorption, renal function, and bone metabolism closely. Recent evidence suggests that adolescents and young females have the highest P demands due to rapid bone growth presumably [22]. Furthermore, age-associated decline in serum P levels also reflects alternation of renal tubular P reabsorption, which was confirmed by results that the median of urinary P excretion in healthy children aged 2–<18 ascended with age [30]. Like the Canadian Health Measures Survey (CHMS), intervals of serum electrolytes were relatively narrow with small fluctuations, consistent with the concept that electrolytes have stable feedback mechanisms throughout life [31].

Collecting samples and establishing pediatric reference intervals can be challenging and costly; therefore, transferability of pediatric reference intervals between laboratories is desired. According to CLSI EP28-A3c guidelines [15], donor laboratory's 95% reference limits may be applied in a receiving laboratory if no more than 2 of 20 test subjects' values (or 10% of the test results) fall outside the original reported limits [15]. The pediatric reference intervals established in the Department of Laboratory Medicine at the First Hospital of Jilin University were verified in five representative hospitals located in different areas of Changchun with the same automatic analyzer and methodology, suggesting they are applicable across Changchun. The automatic analyzers in Lab 1, Lab 2, Lab 3, Lab 4, and Lab 5 are Hitachi 7600-210 automatic analyzer, Hitachi 7180 automatic analyzer, Hitachi 7180 automatic analyzer, Hitachi 7600 automatic analyzer, and Hitachi 7180 automatic analyzer, respectively.

This study established serum electrolyte reference intervals for children aged 2–<14 years and can be applied across Changchun. The reference intervals were quite different with existing Chinese adult standards [8, 10]. The lower limit of our pediatric reference intervals for K was a little higher than adults' (3.5–5.5 mmol/L), and the lower limit of Ca was a little lower than grown-ups' (2.11–2.52 mmol/L). Both the lower and upper limits of Cl were a little lower than adults' (99–110 mmol/L). In contrast, the upper and lower limits of the pediatric reference interval for P are higher than those of adults' standard (0.85–1.51 mmol/L). The lower and upper limits of Na were broader but similar with those of adults' (137–147 mmol/L). These differences indicate that electrolytes are relatively stable throughout lifetime [31]. Unlike Chinese adults, there were sex-specific differences in serum Na, Cl, Ca, and P reference intervals in children aged 13‐14 years, and all serum electrolytes required a minimum of 3 age-specific reference intervals. Therefore, it is important to establish and verify sex- and age-specific reference intervals for prevention, healthcare, and disease evaluation of children. We expect that the reference intervals generated in this study can be directly applied to children living in Changchun and to laboratories using similar instruments and methodology.

Compared with the Abbott Architect c8000 analyzer of CALIPER [21], Abbott Architect ci8200 analyzer of Sweden [32], Roche Modular-P/ISE-system of Denmark [5], and Roche 9180 electrolyte analyzer of India [7], the Hitachi 7600-210 analyzer was used to establish electrolyte reference intervals in the present study. According to the CALIPER study, Ca detected by using the Abbott Architect c8000 analyzer was not transferable to Roche Modular-P [33]. In addition, P results from the Abbott Architect c8000 analyzer correlated only modestly with the Beckman Coulter DxC800 results [34]. Due to the different equipment and methods, the integration and transfer of electrolyte reference intervals need to be further verified.

In our study, serum electrolyte reference intervals for children are different from others. Diet may be an important reason. A study from Mexico City concluded early childhood dietary patterns might play a role in tempo of sexual maturation [35]. Another explanation could be pubertal development, since when sexual dimorphism in tibial bone strength is evident [36] and change in cortical bone density with its distribution differs between boys and girls [37]. The marked effects of puberty on bone metabolism may have obscured any possible effects of diet and vitamin D status [38]. In addition, it cannot be ruled out that the genetic background of Chinese people is different. In brief, the discrepancy among reference intervals for children exists and is critical for evaluating development of children.

## 5. Conclusions

Feasible pediatric reference intervals are lacked in China; thus, we conducted this research to establish K, Na, Cl, Ca, and P ions pediatric reference intervals to fill the gap. Healthy Han children aged 2–<15 years, 646 females and 745 males, were recruited from communities and schools in Changchun, China. Each serum electrolyte had ≤3 age-specific reference intervals. Serum Na, Cl, and Ca showed stable trends within early age groups but fluctuated in teens. The 3 indicators showed no sex-related difference with the exception at age 13‐14, when males' were higher. Serum K and P reference intervals demonstrated complex trends. There were no sex-specific differences for serum K. Serum P reference intervals were highest in children aged 2 years and lowest in children aged 14 years in both sexes. The reference intervals established in our study were verified in subpopulations recruited in five representative hospitals located throughout Changchun and proved to be valid.

## Figures and Tables

**Table 1:** Analytic parameters of electrolytes on Hitachi 7600-210 automatic analyzer.

Ions	Bias of accuracy (%)	Precision				Analytical measurement range (mmol/L)	Clinical reportable range (mmol/L)	Carryover rate (%)
		Low level (%)		High level (%)				
		Within-day	Between-day	Within-day	Between-day			
Potassium	0.71	0.005	0.013	0.081	0.009	2.06–9.09	2.06–9.08	0
Sodium	0.29	0.16	0.53	0.36	0.64	97.1–180.0	97.1–181.0	0.46
Chlorine	1.23	0.39	1.93	0.34	1.46	69.2–120.0	69.2–119.0	0.45
Calcium	−1.00	0.007	0.007	0.024	0.024	1.50–11.25	1.50–11.34	1.14
Phosphorus	1.50	0.90	1.61	1.17	1.78	0.80–1.30	0.60–2.80	1.30

**Table 2:** Sex- and age-specific 2.5th and 97.5th percentiles for electrolyte levels in healthy children aged 2–14 years (*n* = 1391).

Age (year)	Sex	*n*	Potassium (mmol/L)					Sodium (mmol/L)					Chlorine (mmol/L)					Calcium (mmol/L)					Phosphorus (mmol/L)				
			2.5	25	50	75	97.5	2.5	25	50	75	97.5	2.5	25	50	75	97.5	2.5	25	50	75	97.5	2.5	25	50	75	97.5
2-<3	M	109	4.11	4.40	4.60	4.85	5.42	138.4	141.0	142.5	143.8	146.6	99.0	101.4	102.7	104.3	106.3	2.08	2.33	2.39	2.47	2.64	1.37	1.59	1.68	1.76	1.93
	F	87	4.11	4.34	4.54	4.72	5.12	136.1	140.8	142.3	143.4	145.7	98.0	100.4	102.4	103.6	105.6	2.07	2.35	2.43	2.50	2.62	1.39	1.60	1.70	1.76	1.97
3-<4	M	135	3.90	4.37	4.53	4.74	5.41	138.6	140.9	142.3	143.8	147.2	97.9	101.1	102.4	103.6	106.7	1.90	2.20	2.35	2.44	2.56	1.32	1.56	1.67	1.78	2.00
	F	121	3.92	4.27	4.48	4.72	5.26	136.4	141.0	142.4	144.3	147.0	96.2	101.4	102.7	104.0	105.8	2.07	2.29	2.37	2.45	2.56	1.37	1.55	1.67	1.76	1.86
4-<5	M	79	3.79	4.09	4.33	4.64	5.40	136.7	140.3	141.5	143.3	146.4	96.3	100.2	101.4	103.2	105.1	2.04	2.30	2.36	2.45	2.53	1.32	1.51	1.62	1.74	1.94
	F	69	3.70	4.24	4.37	4.62	5.06	137.8	141.2	142.3	143.8	146.4	95.6	100.3	102.2	104.2	105.9	2.09	2.30	2.40	2.47	2.57	1.32	1.51	1.62	1.68	1.86
5-<6	M	78	3.75	4.14	4.38	4.64	5.14	137.7	141.0	142.7	144.3	147.8	98.1	101.1	102.4	103.9	107.0	1.95	2.27	2.35	2.42	2.59	1.36	1.54	1.64	1.75	1.99
	F	75	3.65	4.16	4.35	4.60	4.95	139.1	141.4	142.2	143.7	146.6	98.6	101.4	102.6	104.3	105.8	1.98	2.24	2.36	2.46	2.55	1.27	1.56	1.65	1.73	1.86
6-<7	M	57	3.80	4.27	4.47	4.70	5.17	139.2	141.1	142.2	143.5	145.8	98.2	100.7	102.5	103.4	104.7	1.99	2.21	2.32	2.41	2.61	1.42	1.56	1.65	1.73	2.01
	F	46	3.88	4.26	4.47	4.66	5.02	137.4	140.5	143.0	143.9	145.6	96.9	100.8	102.6	103.8	105.1	2.05	2.28	2.39	2.48	2.56	1.33	1.48	1.59	1.74	1.88
7-<8	M	43	3.70	4.34	4.52	4.67	5.05	138.0	140.6	142.2	144.4	146.8	96.2	99.9	102.1	104.2	106.8	2.11	2.27	2.35	2.40	2.51	1.30	1.50	1.58	1.67	1.98
	F	35	3.82	4.13	4.33	4.63	5.08	138.3	141.3	141.8	143.1	144.7	96.0	101.6	102.9	104.8	107.4	2.17	2.34	2.38	2.47	2.56	1.26	1.48	1.55	1.67	1.87
8-<9	M	32	3.62	4.09	4.42	4.76	5.18	137.8	141.0	142.2	143.7	146.1	97.0	102.0	102.9	104.3	106.2	1.98	2.21	2.28	2.36	2.56	1.26	1.41	1.52	1.62	1.81
	F	23	3.96	4.22	4.38	4.61	5.19	134.3	138.8	140.8	143.2	144.4	97.5	102.3	103.8	107.0	108.2	2.18	2.34	2.38	2.42	2.51	1.30	1.41	1.45	1.51	1.78
9-<10	M	24	3.68	4.18	4.32	4.66	5.06	137.2	140.0	141.6	142.2	144.8	98.5	100.3	101.4	103.2	104.3	2.11	2.26	2.28	2.34	2.42	1.32	1.43	1.50	1.60	1.70
	F	24	3.89	4.10	4.37	4.68	5.21	135.1	137.9	140.2	143.3	145.1	98.6	101.8	103.7	104.8	108.3	2.05	2.28	2.32	2.43	2.59	1.26	1.39	1.52	1.62	1.83
10-<11	M	41	3.78	4.15	4.37	4.59	5.14	137.7	139.7	141.5	143.3	146.4	97.9	100.8	102.6	104.8	108.1	1.98	2.19	2.28	2.37	2.47	1.23	1.38	1.45	1.51	1.69
	F	53	3.79	4.18	4.38	4.55	5.11	138.2	141.8	142.5	143.7	146.4	98.4	102.4	104.5	105.5	106.9	2.07	2.29	2.37	2.46	2.55	1.32	1.41	1.49	1.70	1.85
11-<12	M	29	3.97	4.26	4.51	4.78	5.07	139.6	141.8	143.4	145.1	147.4	102.0	103.9	104.9	105.6	107.7	2.24	2.29	2.33	2.40	2.51	1.38	1.55	1.60	1.66	1.78
	F	27	4.14	4.25	4.39	4.85	5.41	136.8	141.7	143.5	145.0	147.2	98.8	102.3	104.0	106.1	107.4	2.03	2.21	2.32	2.40	2.58	1.36	1.49	1.63	1.72	1.91
12-<13	M	33	4.08	4.35	4.60	4.70	5.19	137.5	140.5	144.8	145.4	149.3	100.5	102.1	103.8	105.4	107.5	2.08	2.26	2.32	2.40	2.50	1.24	1.50	1.60	1.75	2.01
	F	30	4.14	4.34	4.70	5.07	5.34	135.6	139.1	141.4	145.7	147.0	94.52	97.1	99.5	100.9	104.0	2.08	2.17	2.24	2.34	2.44	1.32	1.42	1.48	1.63	1.90
13-<14	M	31	4.15	4.40	4.65	4.92	5.27	138.4	139.8	141.3	142.7	147.2	97.10	99.1	100.6	102.7	105.6	1.96	2.09	2.16	2.24	2.36	1.11	1.36	1.42	1.59	2.03
	F	23	4.17	4.51	4.74	4.95	5.17	134.6	137.8	139.3	140.3	144.4	96.21	98.8	100.7	102.1	106.5	2.03	2.08	2.10	2.14	2.15	1.17	1.30	1.40	1.49	1.61
14-<15	M	54	4.27	4.57	4.82	5.02	5.40	140.2	142.4	144.5	146.0	148.8	98.90	100.9	102.8	104.6	107.1	2.05	2.14	2.20	2.27	2.37	1.09	1.26	1.46	1.59	1.77
	F	33	4.16	4.51	4.67	4.85	5.42	134.8	138.9	140.3	142.5	146.3	96.94	99.8	101.0	102.5	107.0	1.99	2.08	2.16	2.22	2.31	1.14	1.24	1.29	1.36	1.58

M, male; F, female.

**Table 3:** Sex- and age-specific serum electrolyte reference intervals in healthy children aged 2–14 years (*n* = 1391).

Analytes	Age group	Sex group	No. of samples	Lower limit	Upper limit	Confidence interval for lower limit	Confidence interval for upper limit
Potassium (mmol/L)	2 to < 4 years	F + M	452	3.96	5.39	3.89–4.07	5.29–5.47
	4 to < 12 years	F + M	735	3.73	5.27	3.66–3.80	5.14–5.36
	12 to < 15 years	F + M	204	4.14	5.39	4.12–4.18	5.27–5.43

Sodium (mmol/L)	2 to < 9 years	F + M	989	136.4	146.8	134.9–138.2	146.2–147.1
	9 to < 11 years	F + M	142	121.8	146.4	117.8–125.8	145.7–147.1
	11 to < 13 years	F + M	119	136.9	148.6	131.8–142.0	147.3–149.9
	13 to < 15 years	F	56	134.5	146.4	134.1–135.5	145.0–147.6
		M	85	138.6	149.2	138.2–139.9	147.9–149.9

Chlorine (mmol/L)	2 to < 11 years	F + M	1131	94.6	106.4	93.4–96.0	106.0–106.6
	11 to < 13 years	F + M	119	98.4	108.8	95.3–101.5	107.5–110.2
	13 to < 15 years	F	56	93.9	107.4	93.4–96.8	102.6–108.7
		M	85	97.1	107.5	96.6–98.2	105.7–108.9

Calcium (mmol/L)	2 years	F + M	196	2.00	2.64	1.91–2.09	2.60–2.68
	3 to < 13 years	F + M	1054	1.95	2.57	1.91–1.99	2.55–2.59
	13 to < 15 years	F	56	1.96	2.32	1.94–2.08	2.28–2.34
		M	85	1.96	2.37	1.92–2.02	2.34–2.40

Phosphorus (mmol/L)	2 years	F + M	196	1.39	2.65	1.35–1.43	1.96–3.30
	3 to < 8 years	F + M	738	1.32	2.03	1.30–1.34	1.97–2.09
	8 to < 11 years	F + M	197	1.26	1.89	1.22–1.30	1.81–1.97
	11 to < 13 years	F + M	119	1.29	1.91	1.23–1.35	1.81–2.01
	13 to < 15 years	F	56	1.10	1.61	1.07–1.17	1.59–1.63
		M	85	1.00	1.99	0.97–1.05	1.59–1.99

M, male; F, female.

**Table 4:** Validation of electrolyte reference intervals in five laboratories in Changchun.

Analytes	Age group	Sex group	Reference intervals	*N* ^a^	Lab 1		Lab 2		Lab 3		Lab 4		Lab 5	
					*n* ^b^	Result^c^	*n* ^b^	Result^c^	*n* ^b^	Result^c^	*n* ^b^	Result^c^	*n* ^b^	Result^c^
Potassium (mmol/L)	2 to < 4 years	F + M	3.96–5.39	20	0	100	0	100	1	95	1	95	2	90
	4 to < 12 years	F + M	3.73–5.27	20	0	100	0	100	2	90	1	95	1	95
	12 to < 15 years	F + M	4.14–5.39	20	0	100	1	95	0	100	1	95	0	100

Sodium (mmol/L)	2 to < 9 years	F + M	136.4–146.8	20	0	100	2	90	1	95	2	90	1	95
	9 to < 11 years	F + M	121.8–146.4	20	0	100	1	95	2	90	1	95	2	90
	11 to < 13 years	F + M	136.9–148.6	20	0	100	0	100	1	95	1	95	0	100
	13 to < 15 years	F	134.5–146.4	20	2	90	1	95	2	90	1	95	0	100
		M	138.6–149.2	20	1	95	1	95	1	95	0	100	1	95

Chlorine (U/L)	2 to < 11 years	F + M	94.6–106.4	20	0	100	0	100	1	95	0	100	0	100
	11 to < 13 years	F + M	98.4–108.8	20	0	100	1	95	0	100	1	95	1	95
	13 to < 15 years	F	93.9–107.4	20	1	95	1	95	0	100	0	100	1	95
		M	97.1–107.5	20	0	100	0	100	1	95	1	95	1	95

Calcium (mmol/L)	2 years	F + M	2.00–2.64	20	0	100	1	95	0	100	1	95	0	100
	3 to < 13 years	F + M	1.95–2.57	20	0	100	1	95	1	95	0	100	1	95
	13 to < 15 years	F	1.96–2.32	20	2	90	1	95	1	95	1	95	0	100
		M	1.96–2.37	20	1	95	0	100	1	95	0	100	1	95

Phosphorus (mmol/L)	2 years	F + M	1.39–2.65	20	0	100	0	100	1	95	1	95	0	100
	3 to < 8 years	F + M	1.32–2.03	20	0	100	1	95	1	95	1	95	0	100
	8 to < 11 years	F + M	1.26–1.89	20	0	100	1	95	0	100	0	100	1	95
	11 to < 13 years	F + M	1.29–1.91	20	0	100	1	95	1	95	1	95	0	100
	13 to < 15 years	F	1.10–1.61	20	1	95	0	100	1	95	0	100	2	90
		M	1.00–1.99	20	0	100	1	95	0	100	1	95	1	95

M, male; F, female. ^a^Number of validation samples of this study. ^b^Number of validation samples outside the reference intervals of this study. ^c^The results of percentage of validation samples inside the reference intervals of this study.

## Data Availability

The data are stored in the laboratory database.
